# Results of a 4-Year Follow Up of Patients with Paroxysmal and Persistent Atrial Fibrillation after Cryoablation

**DOI:** 10.3390/medicina59112036

**Published:** 2023-11-19

**Authors:** Greta Radauskaite, Gediminas Račkauskas, Svetlana Danilenko, Germanas Marinskis, Audrius Aidietis

**Affiliations:** 1 Department of Cardiovascular Medicine, Vilnius University, 01513 Vilnius, Lithuania; gediminas.rackauskas@santa.lt (G.R.); germanas.marinskis@santa.lt (G.M.); audrius.aidietis@santa.lt (A.A.); 2 Vilnius University Hospital Santaros Clinics, 08661 Vilnius, Lithuania; 3 Department of Mathematical Statistics, Vilnius Gediminas Technical University, 10223 Vilnius, Lithuania; svetlana.danilenko@mf.vu.lt; 4 Department of Human and Medical Genetics, Vilnius University, 01513 Vilnius, Lithuania

**Keywords:** atrial fibrillation, cryoablation, pulmonary vein isolation

## Abstract

*Background and Objectives*: Cryoablation is an established treatment method for atrial fibrillation (AF). We present the long-term results of cryoablation in 94 patients with paroxysmal and persistent AF treated in our center. *Materials and Methods*: This was an observational, retrospective study of 94 patients who underwent a cryoablation procedure for paroxysmal or persistent AF from 2015 to 2017. The follow up was 51 ± 3 months. The absence of arrhythmia was checked at 6, 12, 24, and 48 months after the procedure with 24 h Holter monitoring. We evaluated echocardiography parameters before and 48 months after cryoablation. The quality of life was assessed by calculating EHRA scores at each visit. *Results*: The mean history of pre-procedural AF duration was 55.3 ± 8.6 months. Paroxysmal AF was present in 42% of patients and persistent AF in 58%. Comparing the EHRA classes, a statistically significant difference was observed between the score assessed before the procedure and the score after one year, as well as when comparing the rates before the procedure and four years after the procedure (*p* < 0.000). The recurrence of AF was observed in 22.3% of patients 1 year after the procedure, in 26.6% of patients 2 years after the procedure, and in 34% of patients 4 years after the procedure; 9.3% of them were left in permanent AF. During the observation period, 28% of patients underwent a repeated pulmonary vein isolation procedure, and 6% of patients had a permanent pacemaker implanted. Five hematomas (5%) and one instance of phrenic nerve palsy (1%) were observed during the procedure. *Conclusions*: The rate of arrhythmia recurrence increased every year after cryoablation. Quality of life improved after the procedure, despite the recurrence of AF. A quarter of patients had to undergo a repeat pulmonary vein isolation procedure.

## 1. Introduction

Atrial fibrillation is an increasingly prevalent arrythmia with significant health and socioeconomic impacts [1]. In Europe, AF affects 11 million people, with 886,000 new people being diagnosed each year [2]. AF worsens patients’ quality of life, reduces working capacity, causes heart failure, and increases the incidence of stroke and cardiovascular mortality [2,3]. Symptomatic atrial fibrillation can eventually lead to anxiety disorders and depression. Timely and effective treatment of AF can therefore reduce the cost of treating patients and ease the burden on national healthcare systems. Pulmonary vein isolation (PVI) is an effective treatment for patients with symptomatic paroxysmal or persistent AF [4]. One of the methods of PVI is cryoablation. There is a significantly lower rate of atrial fibrillation recurrence with cryoablation than with antiarrhythmic drug therapy [3]. The 2020 European Society of Cardiology guidelines for the diagnosis and management of atrial fibrillation claim that AF catheter ablation is a safe and superior alternative to antiarrhythmic drug therapy for the maintenance of sinus rhythm and symptom improvement [5].

Some centers report good results one year after the procedure. After 12 months, 82–84% of patients with paroxysmal atrial fibrillation and 70–75% of patients with persistent AF maintained sinus rhythm [6,7]. However, in order to assess the effectiveness of the treatment method, it is important to know the long-term results. Good long-term results could influence changes in treatment tactics, and PVI could become the first-line treatment option for patients with poor tolerance of AF.

There is not much research into long-term results after cryoablation. Most studies are present data for a mean follow-up of about 2 years. Few authors present the results of a longer follow-up. Stenberg et al. published a large prospective cohort of an atrial fibrillation population and the results of a 62-month median follow up. The recurrence rates at 2, 5, and 10 years were 3% vs. 11%, 27% vs. 13%, and 29% vs. 62% for paroxysmal and persistent AF patients [8]. Some other authors have published results for 4-year follow ups, all of which show that recurrence of AF increases every year after the procedure [8,9,10].

We are presenting the results of our 4-year follow up after cryoablation was performed in our center.

## 2. Materials and Methods

### 2.1. Population

This was an observational, retrospective study. We enrolled 95 adults (>18 years of age) who had symptomatic paroxysmal or persistent atrial fibrillation. Paroxysmal AF was intermittent in nature, terminating spontaneously or within 7 days of treatment. Persistent atrial fibrillation was assessed when the rhythm disturbance lasted for more than 7 days. Patients underwent the cryoablation procedure in our hospital between 2014 and 2017.

Indications for procedure were ineffective medical treatment (use of at least one antiarrhythmic drug without a significant effect on the rate of rhythm disturbances), symptomatic paroxysmal or persistent AF, and the patient’s preference to undergo the procedure.

Contraindications were severe heart valve disease, uncontrolled thyroid hyperfunction, or patient refusal to undergo the procedure. All patients were informed about the treatment method, its consequences, and possible complications and signed a consent form for the procedure.

Post-procedural follow-up time was 51.1 ± 3.2 months after cryoablation. We used a 90-day blanking period to assess the results. Patients had visits at one and six months and one, two, and four years after the procedure. A blanking period of 3 months after cryoablation was used because of the difficulty of distinguishing true early recurrences related to peri-procedural reversible causes.

All patients signed an informed consent form for this research. This study was approved by Lithuanian Bioethics Committee.

### 2.2. Cryoablation

A pre-procedural transesophageal echocardiogram was performed to assess for the presence of intracardiac thrombus, and magnetic resonance imaging or computed tomography imaging was performed to review the anatomy of the left atrium and pulmonary veins (PV) before the procedure. Procedures were performed under conscious sedation. After groin puncture, a full dose of heparin was given to reach the activated clotting time >300 s. Transseptal catheterization was performed via the femoral vein under intravascular ultrasound guidance. The transseptal sheath was exchanged for a 15-F Oscor Destino steerable sheath (FlexCath Steerable Sheath; Medtronic, NE Mounds View, MN, USA). A 28 mm diameter second-generation cryoballoon was advanced through the FlexCath sheath into the left atrium and positioned into the antrum of each PV. Ablation was first performed in the left-side veins. The balloon catheter was delivered by using an over-the-wire technique. This delivery was performed by using either a traditional J-tip guidewire or a dedicated cryoballoon inner-lumen octapolar circular mapping catheter (Achieve Mapping Catheter; Medtronic). Adequate PV occlusion with the balloon was determined via injection of a radiopaque contrast agent through the distal end of the catheter. When the inner lumen circular mapping catheter was used, the electrodes were positioned as closely as possible to the PV antrum to monitor for PVI. Typically, a minimum of 2 cryoballoon freezes (each with a duration of 180 to 240 s) was performed at the ostium of each PV. Cryoapplications lasting less than 60 s were classified as aborted ablation attempts and excluded from the analysis. The end point was the electrical isolation of each PV with verification of exit and entrance blocks using the circular mapping catheter.

### 2.3. Patient Follow Up

In our study, patients were followed up at 1 and 6 months and 1, 2, and 4 years after discharge.

We assessed patient demographics such as age and gender. We also evaluated the risk of stroke by calculating a CHA2DS2-VAsc score. We assessed the patient’s clinical condition using NYHA functional class.

At each visit, medical history and an electrocardiogram were obtained. A physical examination was also carried out on each visit. Holter monitoring was performed at 6 months and 1, 2, and 4 years after cryoablation. The recurrence of AF was defined by an electrocardiogram (ECG) or 24 h Holter monitoring of atrial fibrillation or atrial flutter of more than 30 s. The episodes of AF or atrial flutter were treated with antiarrhythmic drug therapy, electrical cardioversion, or the repeated procedure of pulmonary vein isolation/atrial flutter ablation.

We evaluated EHRA score as follows:

- EHRA I—‘No symptoms’;
- EHRA II—‘Mild symptoms’; normal daily activity not affected;
- EHRA III—‘Severe symptoms’; normal daily activity affected;
- EHRA IV—‘Disabling symptoms’; normal daily activity discontinued.

Echocardiographic examination was performed before the cryoablation, 1 year, and 4 years after the procedure. Left atrium (LA) dimensions were measured using 2D methods. We evaluated LA area, LA volume index, LA inferior–superior diameter, and LA medial-lateral diameter in a four-chamber view.

### 2.4. Statistical Analysis

The survey data were processed using SPSS Statistics software (version 26.0. IMB Corp. Armonk, NY, USA).

The quantitative variables were checked for normality using the Shapiro–Wilk test. If the normality condition was confirmed, the means were compared using parametric tests: Student’s t-test was used for 2 independent samples and the Student’s *t*-test for Paired Samples was used for 2 dependent samples. If the normality condition was not met, the non-parametric tests were performed: for 3 or more dependent samples, indicators were compared using the Friedman Test, and the Wilcoxon Signed-Rank Test was performed for 2 independent samples; 2 independent samples were compared using the Mann–Whitney Test. For the qualitative variables, cross-tabulations were calculated. Frequency tables (Crosstab) were produced for the qualitative variables, and the correlation of their attributes was tested with Chi-Square Tests. *p* <0.005 was considered statistically significant.

## 3. Results

There were 94 patients in this study. The main characteristics of patients, as well as their preoperative clinical data, are presented in Table 1.

Patient characteristics are presented according to the type of AF: paroxysmal AF and persistent AF.

The average patient age was 55.29 ± 8.60 years. Duration of AF clinical history was 45 ± 8.28 months. The majority of the population was male, 65 (69.1%). The average patient CHA2DS2-Vasc score was 1.46 ± 1.01. Left ventricular ejection fraction was 54.27 ± 3.78.

Patients with persistent AF had a higher LA volume index than patients with paroxysmal AF. There were no other significant differences between paroxysmal and persistent AF groups.

The procedure time and fluoroscopy time are given in Table 2.

**Figure 1:**
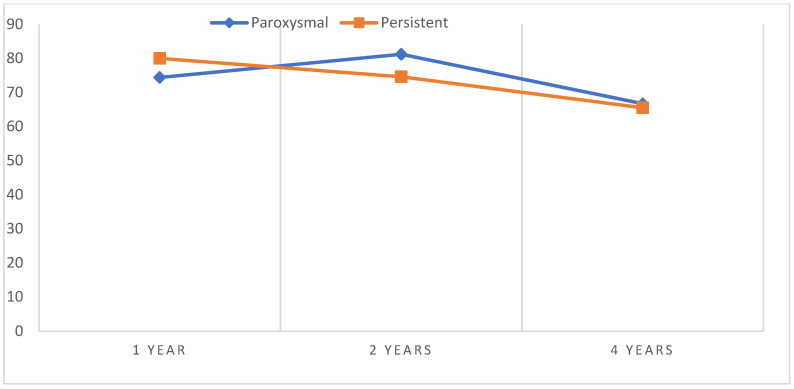
Freedom from arrhythmia over 4 years.

After the cryoablation procedure, freedom from AF was observed in 77.7% of patients 1 year after the procedure, in 73.4% of patients 2 years after the procedure, and in 66% of patients 4 years after the procedure. The sinus rhythm was maintained in 74.4%, 81.2%, and 66.7% of patients with paroxysmal AF and 80%, 74.6%, and 65.5% of patients with persistent AF (*p* = 0.693) (Figure 1). These results show that a large proportion of rhythm disturbances occurred in the first year after cryoablation, with atrial fibrillation recurrences occurring in a smaller proportion of patients in the subsequent period. There was no statistically significant difference between AF recurrence rate and atrial fibrillation type. The arrhythmia recurrence rates were similar in both the paroxysmal and persistent AF groups.

During the observation period, 28% of patients underwent a repeated pulmonary vein isolation procedure and 6% of patients had a permanent pacemaker implanted. Radiofrequency ablation was used for repeat pulmonary vein isolation. Overall, 9% of patients were left with permanent AF, and 2% of them underwent permanent pacemaker implantation and atrioventricular node modification because of tachysystolic atrial fibrillation that cannot be controlled by medication. We did not calculate the recurrence rate of AF after a repeat PVI procedure as we do not have long-term follow-up data. These results could be a continuation of our work.

In total, five hematomas (5%) and one phrenic nerve palsy (1%) were observed during the procedure. All hematomas were minor, not requiring surgical or conservative treatment. Phrenic nerve palsy was asymptomatic. None of the complications prolonged the patients’ hospitalization, affected their quality of life, or left any residual effects.

We evaluated the left atrial parameters before and 4 years after cryoablation in two groups: patients with recurrence of AF and patients with sinus rhythm (Table 3).

Patients with post-procedure atrial fibrillation had a higher left atrial area than those without AF (26.22 ± 3.88 vs. 24.35 ± 3.91, respectively; *p* = 0.022). The LA medial–lateral diameter was also larger in patients with recurrence of AF (49.5 ± 5.2 vs. 46.8 ± 4.8, respectively; *p* = 0.019). There was no significant difference in the other left atrial parameters between the two groups.

**Figure 2:**
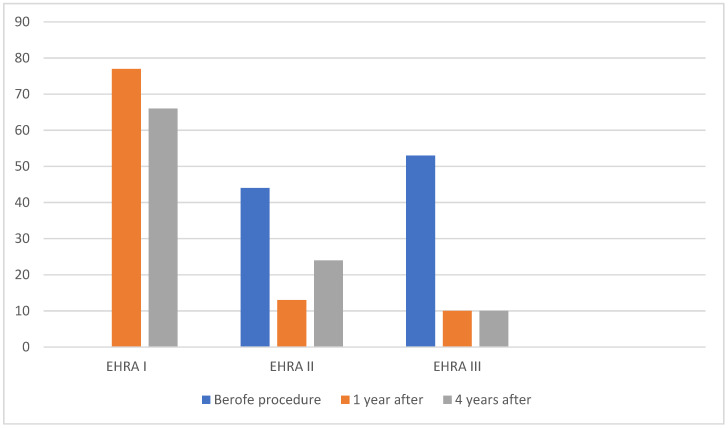
EHRA score before cryoablation, 1 year, and 4 years after procedure.

We also assessed quality of life by calculating the EHRA score before the procedure and at each follow-up visit. Before the cryoablation procedure, 41 patients (44%) had EHRA II scores and 53 patients (56%) had EHRA III scores. There were no patients with an EHRA I score. One year after the procedure, 73 patients (77%) had EHRA I scores, 12 patients (13%) had EHRA II scores, and 9 patients (10%) had EHRA III scores. Four years after the procedure, 62 patients (66%) had EHRA I scores, 23 patients (24%) had EHRA II scores, and 9 patients (10%) had EHRA III scores (Figure 2). When comparing the EHRA scores, a statistically significant difference was observed between the rate assessed before the procedure and the rate after one year, as well as when comparing the rates before the procedure and four years after the procedure (*p* < 0.000). There was no statistically significant difference when comparing the EHRA functional scores one year after the procedure and four years after the procedure (*p* = 0.180). These data show that cryoablation significantly improves quality of life and reduces AF symptoms in patients. The effect on quality of life was long-lasting and persisted after four years. Symptom reduction was observed even in those with recurrent AF.

## 4. Discussion

There are several possible treatment methods for atrial fibrillation. Medical therapy is the first choice, but its effectiveness in reducing the frequency and symptoms of AF recurrence is limited [3]. Ablation is recommended as a second-line AF treatment therapy when medical treatment is ineffective or not tolerated in patients with paroxysmal or persistent AF [5,11,12]. Radiofrequency (RF) ablation and balloon cryoablation can be used to achieve pulmonary vein isolation [13]. However, some studies show better outcomes and lower complication rates in patients after cryoablation than after RF [8,11,13,14]. Also, cryoablation significantly shortened the procedure time, nonetheless, with negligible impact on the fluoroscopy time [15]. The current results suggest that cryoablation is a safer treatment option than radiofrequency ablation. However, new approaches to the treatment of AF are coming into clinical practice and may change the current treatment tactics, such as pulse field ablation. There are no long-term follow-up results for this treatment method yet, but published one-year results are promising [16]. The PULSED AF pivotal study confirmed a low rate of serious procedure-related adverse events, a high rate of freedom from atrial arrhythmia recurrence, a clinically significant improvement in quality of life from baseline, and efficient procedure times with the use of pulsed field ablation technology [16]. Ultimately, future multicenter randomized trials will be necessary to accurately characterize relative safety and efficacy compared to conventional thermal ablative strategies to pulse field ablation. The results of such trials may implicate some of the effects of current atrial fibrillation guidelines as well.

Major complications after cryoablation include pericardial effusion/tamponade, embolic events, pulmonary vein stenosis, atrioesophageal fistula, phrenic nerve palsy, and vascular access problems such as hematoma, retroperitoneal bleeding, femoral pseudoaneurysm, or femoral arteriovenous fistula [13,17,18]. The complication rate has been reported to be between 2.2 and 7.0% [13,17]. We observed two types of complications: phrenic nerve injury and hematomas. The rate of vascular access site complications is reported to be between 1.1 and 6.3% in other studies [17], with that of phrenic nerve injury being 2.2–6.38% [13,17,19]. The rate of complications in our center was very similar to those reported in other centers: 5% of patients had hematomas and 1% had phrenic nerve palsy. E. Ströker et al. provided important data in terms of ultrasound-guided venipuncture, which was associated with a near-to-zero risk of vascular complications in patients undergoing cryoballoon ablation for atrial fibrillation [20]. A meta-analysis involving 8232 patients in nine studies was presented in 2019 [21]. Data presented in this meta-analysis compared ultrasound-guided vs. palpation-based techniques for femoral venous access in electrophysiology procedures. Compared with the standard technique, the use of ultrasound reduced major vascular complications (from 2.01 to 0.71%, *p* < 0.0001). The rate of minor vascular complications (RR = 0.30, 95% CI, *p* = 0.001) and inadvertent artery puncture were lower with ultrasound-guided puncture (RR = 0.31, 95% CI, 0.17–0.58, *p* = 0.0003). A subgroup analysis of patients undergoing PVI also showed a significant reduction in major vascular complications (RR = 0.27, 95% CI, 0.12–0.64, *p* = 0.003) [21]. We used vascular ultrasound to guide venous femoral access in all cases. We think that this is why we did not observe patients with major vascular complications such as pseudoaneurysms or femoral arteriovenous fistulas. However, the formation of hematomas also depends on the anticoagulant therapy used and the optimal hemostasis [17]. In our center, patients discontinued NOACs 24 h before the procedure. Patients on warfarin discontinued 72 h prior to the procedure using bridge therapy with low-molecular-weight heparin. After groin puncture, a full dose of heparin was given to reach activated clotting time > 300 s. Reversal of heparin-mediated anticoagulation via protamine sulfate prior sheath removal has been shown to be useful for the prevention of vascular access complications in patients undergoing cryoablation for AF, but we use it in case of major bleeding complications [17]. None of the observed post-procedural complications prolonged the length of hospital stay of patients. The hematomas were local, small in diameter, and did not require treatment. Damage to the phrenic nerve was also asymptomatic, transient, and regressed by the time of discharge from the hospital.

The effectiveness of cryoablation is measured in terms of freedom from AF. This indicator varies depending on the center, from 60 to 82% during the first year after cryoablation [3,6,14,22], and 48–54% four or more years after the procedure [8,23]. Several mechanisms (atrial local inflammation, increased adrenergic tone, and changes in fluid and electrolyte balances) play a role in determining the transient increase in the risk of post-procedural atrial tachyarrhythmias occurring early after ablations. Therefore, a blanking period of 3 months after any form of PVI has been accepted because of the difficulty of distinguishing true early recurrences from transient early recurrences of arrhythmia related to peri-procedural reversible causes [24]. Heeger et al. reported the five-year clinical outcomes following second-generation cryoballoon-based pulmonary vein isolation in patients with paroxysmal and persistent atrial fibrillation. They found an overall single-procedure 5-year clinical success rate of 54% (59% in paroxysmal AF and 44% in persistent AF) [25,26]. Akkaya et al. described a single-procedure success rate after ablation of 61% in paroxysmal AF and 52% in persistent AF [27]. Our early results are comparable to those other centers, and our long-term results look slightly better: 66% of patients had freedom from arrhythmias (66.7% of patients with paroxysmal AF and 65.5% of patients with persistent AF). Also, we found that about two-thirds of all AF recurrences occurred in the first year after cryoablation. Some authors describe similar results [28]; others claim an AF recurrence of more than 50% during the first year after the procedure [10]. These results suggest that patients without early arrhythmic episodes have better cryoablation outcomes.

Atrial fibrillation disrupts patients’ normal rhythm of life and has a significant impact on quality of life. Studies show that cryoablation significantly improves quality of life compared to drug therapy [29,30]. Freedom from AF most often resulted in a reduction of symptoms, but symptom relief also occurred despite little effect on the arrhythmia [31,32]. Improvements in symptoms and quality of life have been observed even in patients with recurrent AF [32]. AF ablation may change the perception of AF so that patients may experience diminished symptoms despite recurring episodes of arrhythmia [29]. The CABANA trial demonstrated that catheter ablation led to significant improvements in quality of life that were maintained at 5 years after ablation when compared with medical therapy [33]. Depression is also closely related to the occurrence and development of AF, which increases the complexity of management and the risk of adverse outcomes in patients with AF. The studies found that the incidence of depression was significantly higher in patients with AF than that in patients without AF [34]. Depression is associated with frequent AF recurrence. However, there are no data yet on the effect of cryoablation treatment on depression. This study could provide new directions and stimulate further development of research on cryoablation for AF and depression.

Several prognostic factors for AF recurrence are described. Among the most frequently mentioned are the duration of AF before cryoablation, LA diameter, and type of AF [9,33]. No difference in AF recurrence between paroxysmal and persistent AF patients was observed in our study. The rate of AF recurrence was also independent of the duration of AF before the procedure. However, arrhythmias were more frequent in those with larger LA diameter. More significant atrial structural and electrical remodeling may explain the predictive role of LA diameter for AF recurrence [33]. Some studies describe that a decrease in LA appendage emptying velocity is also associated with the recurrence of AF [10]. More detailed multicenter studies with a larger number of patients would probably be needed to more accurately assess the significance of clinical, anatomical, and demographic patient characteristics to AF recurrence rates. These multicenter studies could modify the indications according to the identified prognostic factors and adjust the guidelines.

The limitations of our study are as follows: this study is one single-center experience. It is a non-randomized trial. Some patients were lost to surveillance due to the COVID-19 pandemic. Atrial fibrillation paroxysms were detected by ECG and Holter monitoring, so some asymptomatic relapses may have been missed. 

## 5. Conclusions

Cryoablation is a safe and effective method of treating atrial fibrillation, especially for those with severe symptoms. It improves quality of life and reduces AF recurrence, although this result decreases with time.

## Figures and Tables

**Table 1:** Patient characteristics.

	Paroxysmal AF (=39)	Persistent AF (*N* = 55)	*p*-Value
Age	54.38 ± 8.41	56.50 ± 7.73	0.213
Female gender	14	15	0.372
CHA2DS2-VAsc Score 0	7	8	0.96
CHA2DS2-VASc Score 1	15	23	
CHA2DS2-VAsc Score 2	12	16	
CHA2DS2-VASc Score ≥3	5	8	
NYHA functional class I	24	32	0.940
NYHA functional class II	12	12	
NYHA functional class III	3	5	
LA area (cm^2^)	25.42	25.33	0.117
LA volume index (ml/m^2^)	34.58 ± 9.23	40.19 ± 9.32	0.018
LA inferior–superior diameter (mm)	58.2 ± 7.9	59.5 ± 6.7	0.112
LA medial–lateral diameter (mm)	44.8 ± 6.1	51.1 ± 3.3	0.058
AF duration (months)	41.95	48.91	0.214

**Table 2:** Characteristics of the procedure.

	Average	Standard Deviation
Procedural time (min.)	90.22	±39.71
Fluoroscopy exposure (min.)	13.45	±8.69
The X-ray dose (mGy/m²)	429.59	±204.00

**Table 3:** LA characteristics of AF recurrence and no-recurrence patients before procedure and 4 years after.

	Recurrence of AF	No Recurrence of AF	*p*-Value
LA area (cm^2^) before	24.59 ± 5.00	25.25 ± 3.70	0.663
LA area (cm^2^) after	26.22 ± 3.88	24.35 ± 3.91	0.022
LA inferior–superior diameter (mm) before	60.1 ± 7.8	58.3 ± 6.4	0.332
LA inferior–superior diameter (mm) after	62.0 ± 4.7	61.0 ± 5.5	0.092
LA medial–lateral diameter (mm) before	45.1 ± 5.1	45.7 ± 5.2	0.587
LA medial–lateral diameter (mm) after	49.5 ± 5.2	46.8 ± 4.8	0.019
LA volume index (mL/m^2^) before	39.8 ± 9.2	40.6 ± 7.1	0.672
LA volume index (mL/m^2^) after	40.2 ± 9.7	42.0 ± 7.1	0.652

## Data Availability

The data presented in this study are available on request from the corresponding author. The data are not publicly available due to ethical requirements.
